# TISSUE GRAFTS IN VITILIGO SURGERY – PAST, PRESENT, AND FUTURE

**DOI:** 10.4103/0019-5154.53196

**Published:** 2009

**Authors:** Niti Khunger, Sushruta Dash Kathuria, V. Ramesh

**Affiliations:** *From the Department of Dermatology and STD, V.M. Medical College and Safdarjang Hospital, New Delhi, India*

**Keywords:** *Vitiligo surgery*, *tissue grafts*, *suction blister grafting*, *thin split thickness grafting*

## Abstract

Vitiligo, characterized by depigmented macules is a common disorder with a high psychosocial impact, particularly in darker skins. Surgical methods become important in cases where medical therapy fails to cause repigmentation or in cases of segmental vitiligo where the response to surgery is excellent. The basic principle of surgical treatment is autologous grafting of viable melanocytes from pigmented donor skin to recipient vitiliginous areas. Various grafting methods have been described including tissue grafts and cellular grafts. Stability of the disease is the most important criterion to obtain a successful outcome. Counseling of the patient regarding the outcome is vital before surgery. The technique and followup management of the tissue grafts has been described in detail in this review.

## Introduction

Vitiligo is a common depigmenting disorder, characterized clinically by milky white macules and histologically by an absence of functional melanocytes in the affected area. It causes severe cosmetic distress, particularly in darkly pigmented skins and is also associated with a great social stigma. It has a profound psychological impact and greatly affects the quality of life.[1] In addition, the depigmented skin becomes photosensitive on the exposed areas of the skin, leading to redness and burning on sun exposure. Depending on the type, extent, and duration of vitiligo, conventional medical therapies such as topical and systemic corticosteroids, topical immunomodulators, and phototherapy are not always successful, and repigmentation is often incomplete. Vitiliginous lesions occurring on sites such as lips, acral areas, nipples, and genitals are particularly resistant to medical treatment. This has led to the evolution of various surgical modalities to treat recalcitrant stable lesions. The conventional surgical modalities for vitiligo are miniature punch grafting, suction blister grafting, and thin split thickness skin grafting. Recent advances include autologous noncultured epidermal cell suspensions and cultured melanocyte suspensions or sheets.

## Historical Aspects[2]

Skin grafting was first described in India in ancient Sanskrit texts around 2500 - 3000 BC as a technique for nasal reconstruction for mutilated noses. Thin split thickness skin grafts were first introduced in 1872 by Ollier in France, and later by Thiersch in Germany in 1874. Brown in England developed the electric dermatome in 1944, to harvest thin homogenous grafts. In 1947, Haxthausen transplanted thin split thickness skin grafts from normal to vitiliginous skin in three cases, to study the pathogenesis of the disease.[2,3] In 1964, Behl from India was the first to describe the surgical treatment of vitiligo in a large series of 107 patients with thin Thiersch grafts.[4] Falabella described the suction blister technique for repigmentation of vitiligo in 1971[5] and later the miniature punch grafting technique in 1978.[6] Falabella *et al*.,[7] in 1989, also described the use of *in vitro* cultures of melanocyte-bearing epidermis for the treatment of vitiligo. The use of epidermal suspensions obtained by trypsinization was first reported in 1992 by Gauthier and Surleve-Bazeille[8] and further improved by Olsson and Juhlin,[9] by adding a melanocyte culture medium, for additional growth. Kahn and Cohen[10] utilized the motorized dermatome, to obtain ultrathin grafts for vitiligo, and later in 1996 Kahn *et al.*,[11] reported the use of a short-pulse carbon dioxide laser, to denude the recipient area. Subsequently, the excimer laser and targeted phototherapy have been developed to treat vitiligo. Thus, surgical treatment of vitiligo has evolved over the centuries, even though the etiology and pathogenesis of vitiligo remain elusive.

## Basic Principles

The basic principle of surgical treatment in vitiligo is to achieve cosmetically acceptable repigmentation of the vitiliginous areas by transplantation of autologous melanocytes from the unaffected pigmented skin to the lesional skin. It cannot stop the progression of the disease, and is indicated for resistant stable vitiligo that does not show adequate response to medical therapy. Different surgical modalities are available and the choice of surgical treatment depends on the type of vitiligo, extent and site of the lesions, and the availability of equipment and expertise of the treating surgeon.

## Concept of Stability in Vitiligo

Stability of the disease process in vitiligo is the most important parameter to achieve a successful outcome in surgical treatment. Stability is defined as the absence of new lesions and absence of the spread of existing lesions for a defined period. However, there is no consensus on the period of stability, and it varies from 4 months to 2 years, according to different authors.[12– 15] A recent publication on the standard guidelines of care in vitiligo, suggested 1 year as an acceptable period to establish stability, with the following definition; a patient reporting absence of new lesions, absence of progression of existing lesions, and absence of Koebner phenomenon in the previous one year.[16] Since the history provided by the patient may not be entirely reliable, other methods of establishing stability have been proposed, such as, test grafting and Vitiligo Disease Activity Score (VIDA) scoring. Falabella *et al*.,[17] proposed the test graft method which consisted of placing six to eight punch grafts within a vitiligo lesion and observing the repigmentation over the next 12 weeks. Unequivocal repigmentation occurring beyond 1 mm from the border of the test graft indicates a positive test and is taken as an indicator of stability. However, doubts have been expressed over its utility as it has been seen that even when the disease may be unstable the minigraft test is positive and also the test may only confirm the stability of the lesion tested and not necessarily of the disease process in the patient.

Njoo *et al*.,[18] proposed the VIDA as another method of establishing objective criteria for stable case selection. It is a six-point scale on which the activity of the disease is evaluated by the appearance of new vitiligo lesions or the enlargement of the pre-existing lesions, gauged during a period ranging from less than 6 weeks to one year. It is suggested that surgery for vitiligo may be performed only in patients with VIDA scores of -1 or 0.

## Indications

Surgery in vitiligo is indicated in patients with stable vitiligo not responding to medical treatment or causing severe psychosocial distress. It can also be performed in patients with leukoderma due to burns, piebaldism, inactive discoid lupus erythematosus, and other stable disease states causing permanent depigmentation.

## Contraindications

Vitiligo surgery is contraindicated in patients with active unstable vitiligo and in childhood vitiligo. In children, progress of the disease is difficult to predict and by and large they respond better to medical therapy as compared to adults. In addition, surgery has to be carried out under general anesthesia, which is another added risk factor in children.

## Procedures

There are various surgical modalities to treat vitiligo, the goal being to achieve complete repigmentation; cosmetically matching the surrounding normal skin. The choice of surgical treatment depends on the extent of vitiligo, the sites involved, the availability of equipment, and the expertise of the surgeon. Various surgical methods including tissue grafts and cellular grafts are described in Table 1.

**Table 1:** Grafting techniques in vitiligo

Grafting techniques
*Tissue grafts*	*Cellular grafts*
Minipunch grafting	Noncultured basal cell suspensions
Suction blister grafting	Cultured melanocytes/keratinocyte grafts
Thin split thickness
grafting
Hair follicle grafts
Mesh grafts
Flip-top pigment
transplantation

## Tissue Grafts

### Mini punch grafting

Mini punch grafting has been discussed in detail in a separate article.

### Suction blister grafting

Suction blister grafting (SBG) is a technique where the pigmented epidermis is harvested from the donor site by using suction to raise a blister which is then transferred to the vitiliginous area.

## Principle

The differentiation and development of the epidermal cells is regulated by the dermis. In surgical techniques like split skin thickness grafting and punch grafting, where both the epidermis and dermis are grafted, the graft retains some of the characteristics of the donor site, hence, the cosmetic outcome may not be an exact match. However, in suction blister grafting, cleavage occurs between the basal cells and the basal lamina of the basement membrane zone and only the epidermal portion of the donor area is grafted. Hence, the graft generally acquires the characteristics of the recipient site, thus leading to a better color match and cosmetic outcome.

## Technique

### Harvesting of the graft site

The donor site can be anywhere from the flexor aspect of the arm or forearm, abdomen, or the anterolateral aspect of the thigh or leg. In a study by Laxmisha and Thappa,[19] it was found that there was 100% success in raising blisters on the flexor aspect of the arm, and complete blisters were most often raised on the leg or flexor aspect of the forearm; although the number of patients was small. However, covered sites such as the gluteal region or the thigh are preferred, as pigmentary changes can occur at the donor site.

### Preparation of donor site

After surgical cleansing, a topical local anesthetic may be applied as the procedure is painful. Some prefer injecting the area with local anesthetic with saline as it reduces blister induction time (BIT).

### Raising of blisters

**Figure 1:**
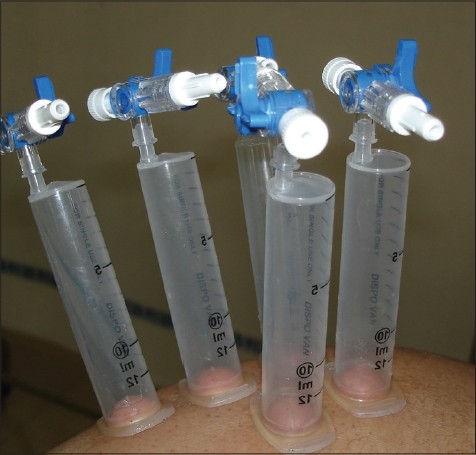
Raising suction blisters by the syringe method

**Figure 2:**
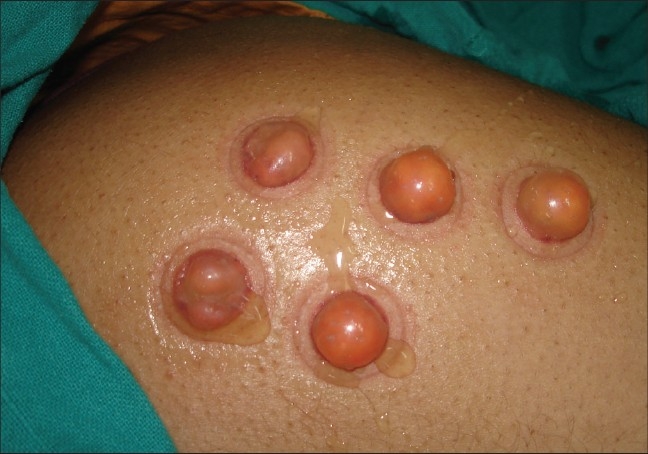
Blisters formed after 2 hours of suction

Blisters may be raised using syringes[20] or suction pump and suction cups[21] or a negative pressure cutaneous suction chamber system.[22] Using syringes to raise blisters is the most commonly employed method. The bases of syringes of sizes 10 ml and 20 ml are coated with vaseline and are applied on the donor site [Figure 1]. Approximately 20ml to 30ml of air is aspirated using a 50ml syringe and a three-way-cannula. Larger syringes (>20ml) take a longer time to generate a blister, and smaller syringes (<10ml) provide blisters that are difficult to handle. It usually takes 1.5 to 2.5 hours for the development of blisters [Figure 2]. A single unilocular non-hemorrhagic blister is the best result. In case of smaller blisters, one can either increase the negative pressure in the syringe by another 5ml or intradermally inject saline into the blister to expand it.[23]

### Deroofing the blister

Once the blisters are well formed, the roofs of the blisters are gently cut using iris scissors. Care should be taken to avoid touching the floor of the blisters as it causes pain. The roofs are inverted onto a glass slide such that the dermal side faces upwards. The graft is then cleaned and spread to its maximum size and kept moist with normal saline. The donor site is cleaned and bandaged using nonadherent dressing such as chlorhexidine gauze (Bactigras^®^).

## Transfer of Graft

The vitiliginous macule is surgically cleaned using spirit and povidone iodine and then anesthetized using plain lignocaine 1%. The area can be dermabraded using a manual dermabrader, motorized dermabrader, microdermabrader or a CO_2_ laser till minute pinpoint bleeding spots are visible. Liquid nitrogen freezing or suction blistering[24] have also been used for the preparation of the recipient site. The grafts are then placed such that the dermal side of the graft is now in contact with the dermabraded area. A gap of 0.5cm can be left between two grafts because there is a pigment spread. A nonadherent dressing is applied and then bandaged using Dynaplast^®^. It has been considered that preparation of the recipient site by suction blistering may be superior to the CO_2_ laser as the graft is better taken up and repigmentation is faster.[25]

## Postoperative Care

The dressing over the donor site is removed after 24 hours and cleaned daily. The dressing over the recipient site is left on for 7 days. The patient is advised to keep the area immobile. Usually, the grafts fall off in 1 to 2 weeks; so essentially this is a technique of melanocyte transfer. Very rarely are the grafts taken up.

## Follow-up

**Figure 3:**
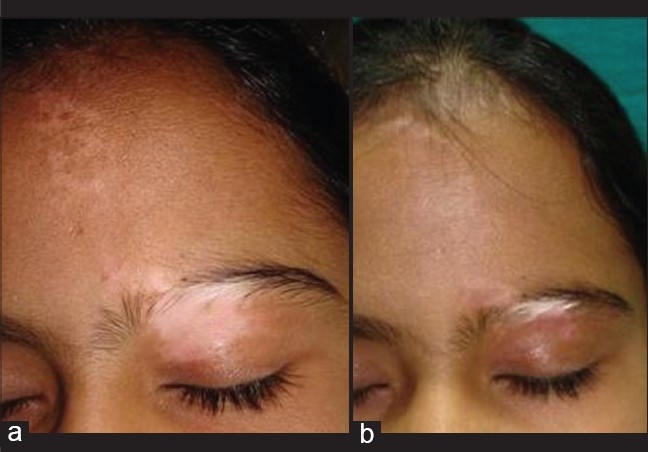
a) Stable resistant zosteriform vitiligo, b) Repigmentation 3 months after suction blister grafting with persistence of leukotrichia

The patient may be started on oral or topical Psoralen-UVA or PUVASOL from the day of removal of dressing. Repigmentation usually occurs in 3 month's time [Figure 3]. Rarely the leucotrichia may also repigment.

## Complications

Complications are uncommon, although hyperpigmentation, incomplete pigmentation, perigraft halo, and graft rejection may occur.

## Efficacy

In a meta-analysis, it was found that 87% of the patients achieved more than 75% repigmentation with SBG, which was similar to split thickness grafting (87%), but better than minigrafting (68%) and grafting of noncultured epidermal suspensions(31%).[26] Segmental and focal vitiligo responded better (91% achieving > 75% repigmentation) than generalized vitiligo (53%, *P* <.001). There was no significant difference in repigmentation rates at different body sites.[27]

## Advantages

It is a safe, easy, and inexpensive method, with very good success rates. Repigmentation is faster and the color match is very good, especially over the lips,[28] eyelids[29] and areola. One group observed that the color match was better with punch grafting over the lips as compared to SBG, however, the number of patients were small.[30] Scar formation, keloid, cobblestoning, sinking pits, thick margins, and milia formation was much less compared to split thickness grafting or punch grafting.[26]

## Disadvantages

It is time consuming and the raising of blisters is painful. Larger areas require multiple sittings. Improper handling may lead to tearing of the graft or the epidermal side being grafted, causing failure of repigmentation.

## Split Thickness Skin Grafting

In this technique, thin split thickness skin grafts are harvested from the pigmented donor area and transplanted at the recipient sites as continuous sheets of tissue grafts.

## Principle

In the surgical treatment of vitiligo, thin split thickness grafts are used. There are three biological changes which follow skin grafting.[31]

### Graft take adherence

There are two phases in this stage. In the first 72 hours of the placement of the graft adherence of the graft is due to fibrin bonding and the graft appears pink. Hence, these first 72 hours are most crucial for graft uptake and bleeding, infection, and mechanical movement due to improper immobilization of the area can lead to graft failure at this stage. Hence strict immobilization in the first three days is very essential. The second phase begins with the onset of vascular anastomosis and fibrovascular growth.

### Graft revascularization

In this stage there is a connection between the graft and host vessels, with the formation of new vascular channels. Insufficient vascular proliferation, development of a thick layer of fibrin or hematoma or seroma can lead to failure of graft uptake in this stage. Hence compression is useful.

### Contracture

There is contracture of the graft when it is harvested because of the contraction of the elastin fibers. Contracture also occurs at the recipient site. These two factors lead to achromic fissures and perigraft halo. Overlapping of graft edges at the recipient site can prevent these complications.

## Technique

### Harvesting of the graft

**Figure 4:**
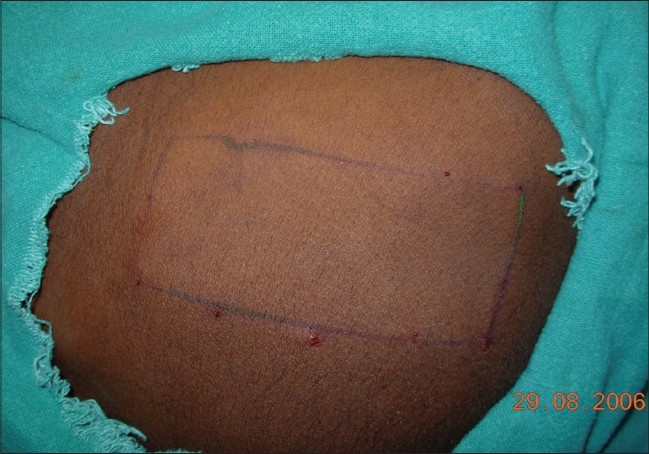
Infiltration anesthesia at the margins of the donor area without infiltration in the centre

Though the gluteal area is a relatively difficult area to harvest, it is the most preferred site for cosmetic reasons, in case scarring occurs. However, the thigh can be used as a donor site by beginners and when larger areas of donor skin are required.[31] The arms have also been used as donor sites.[32] After surgical cleansing, the required donor area is marked with sterile marking ink or a surgical pen. A field block with 1% lignocaine is given at the margins only [Figure 4]. Infiltration anesthesia within the donor area must be avoided as it can result in an uneven surface, leading to a graft of uneven thickness. Alternatively, topical anesthesia using a mixture of lidocaine 2.5%w/w and prilocaine 2.5% w/w cream (EMLA^®^, Toplap^®^) under occlusion, for at least two hours, can be used.[33]

**Figure 5:**
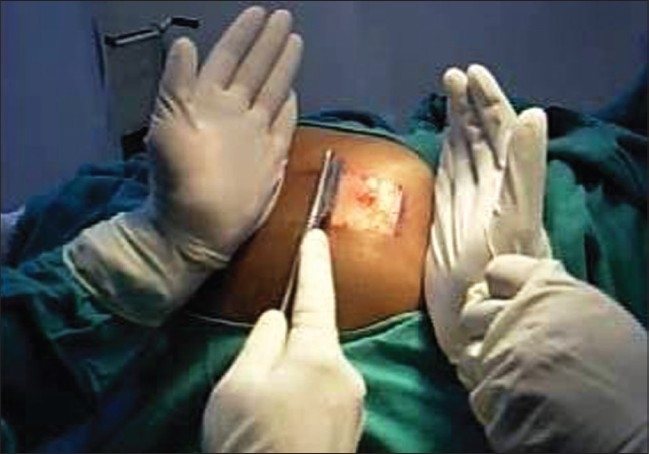
Harvesting a thin split thickness graft freehand with a sterile razor blade mounted on a Kocher's forceps

The skin is stretched firmly at one end by an assistant with the flat of the hand or a wooden block and the other end is stretched by the operator. A thin even split thickness graft is harvested free hand using either a sterile razor blade mounted on a Kochers forceps or a blade holding instrument [Figure 5]. Alternatively, a hand dermatome, Humby's knife[34] or Silvers knife[35] may be used, depending on the expertise of the surgeon and availability of the instruments. However, these techniques require skilled operators to harvest thin, even grafts. Of late, an air driven power dermatome (Zimmer Inc. Warsaw Ind USA) is being used to harvest ultrathin epidermal sheets.[14] The dermatome is fitted with a special shield plate to control the size and thickness of the graft.

The donor skin is kept in a sterile petri dish containing normal saline and the donor area is dressed with nonadherent dressing (Chlorhexidine dressing Bactigras^®^ or Tegaderm 3M, St. Paul, Minn) and a pressure dressing is given. The dressing is left undisturbed for a week.

## Transfer of Graft

**Figure 6:**
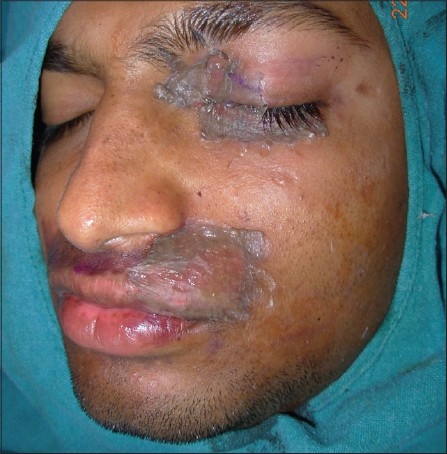
Cyanoacrylate adhesive applied to secure the graft

The recipient area is anesthetized by using either a topical anesthetic cream applied under occlusion 2 to 3 hours before the procedure by the patient, or infiltration anesthesia using 1% lignocaine without adrenaline is administered. For larger areas, general anesthesia is usually required. Preoperative medication, diazepam, 10mg orally at night and 2 hours before the operation relieves anxiety and may be given in anxious patients. After surgical cleansing, the vitiliginous area is first marked with a surgical pen and abraded by dermabrasion with a diamond fraise attached to an electric high-speed dermabrader at 10,000 rpm till pinpoint bleeding is seen. The recipient site can also be prepared using a pulsed Erbium-YAG laser or ultrapulse CO_2_ laser. Kahn *et al*.,[11] reported no histological difference in skin graft adherence between the short pulsed CO_2_ laser and a dermabrader. The ultrapulse CO_2_ laser is preferred over the Er:YAG laser because it achieves better hemostasis and causes an epidermal - dermal split at a single pass.[36] Manual dermabrasion with a manual dermabrader may also be used by beginners or when there is lack of equipment. The graft is carefully placed over the denuded recipient site, taking utmost care to place the dermal surface facing down. Immobilization of the graft is most important and is achieved by using surgical adhesive, octyl-2-cyanoacrylate and pressure dressing [Figure 6]. The adhesive gives excellent results to secure the graft and also has antimicrobial properties against staphylococci, pseudomonas, and *E. coli.*[37] In a study of 50 patients of stable, recalcitrant vitiligo, treated with split thickness skin grafting of over 180 lesions, cyanoacrylate adhesive was effective in immobilizing the grafts especially over the mobile areas.[38] It is best applied in an interrupted manner at the edges of the graft to allow drainage of serous fluid and at the center over mobile areas such as eyelids. A nonadherent dressing is then applied.

## Postoperative Care

The first dressing is preferably changed at 24 hours, to observe for any serous collection or hematoma under the graft, which can be drained. Subsequently, the dressing is changed after 1 week by which time healing is almost complete. Prophylactic oral antibiotics are given for one week to prevent postoperative infection.

## Follow-up

If there is perigraft depigmentation or achromic fissures, NB- UVB is required postoperatively for complete repigmentation.

## Efficacy

Thin split thickness skin grafting is the most successful technique among all the surgical methods, with a success rate of 78 - 91%.[26]

## Complications

Hyperpigmentation is the commonest complication in dark skinned patients and takes a long time to resolve.[31,39] Peripheral depigmentation (halo) at the edges of the grafted area and achromic fissures between the grafts are common due to graft shrinkage. These respond to treatment with topical or systemic psoralens or NB-UVB. To avoid this complication, the graft should extend 1 - 2 mm beyond the abraded area. Milia are also frequently seen. They resolve spontaneously or can be expressed out. Hypertrophy of the graft occurs in thicker grafts. It usually subsides spontaneously over a period of 3 to 4 months, but may persist for longer periods. Scarring at the donor site can occur in inexperienced hands. Secondary infection is uncommon.[40] Recurrence of vitiligo at the grafted or donor site or appearance of new lesions may occur in unstable cases, or if the disease becomes reactive after a period of stability.

## Advantages

The advantages of split thickness skin grafting are that pigmentation can instantly cover larger areas over a short period of time as compared to other techniques. Difficult areas such as eyelids, inner canthus of eyes, areola, nipples, and genitals can be treated readily. Pigmentation achieved is uniform and cobblestoning that is common with punch grafting is not seen. Repigmentation of leukotrichia is also possible.[34] In addition, as compared to noncultured or cultured melanocyte suspensions, no reagents, laboratory facilities or expensive elaborate equipment are required.

## Disadvantages

Prolonged hyperpigmentation is commonly seen, particularly on the exposed areas in dark-skinned patients. Large areas require multiple sittings due to limitation of the donor site. Surgical skill is required to take thin even grafts free-hand. However, the recent use of electric dermatomes has made it easier to harvest uniform ultrathin grafts. In addition, the use of Er:YAG or CO_2_ laser to prepare the recipient areas reduces blood splatter as compared to dermabrasion and are further advances in the technique.

## Modification of Techniques at Special Sites

Certain precautions and modification of techniques can optimize results at sites that are difficult to treat.

### Eyelids:

While grafting the upper eyelid the thinnest graft should be selected. If the area is small, a suction blister graft is ideal. When almost the entire eyelid is involved, thin or ultrathin split thickness skin grafts give optimum results. Strict immobilization for the first 72 hours by application of cyanoacrylate adhesive and pressure bandage is essential for graft uptake.

### Lips:

**Figure 7:**
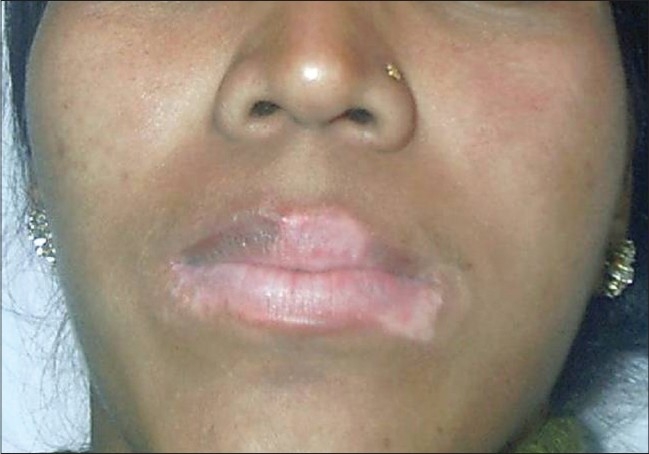
Stable vitiligo of the lips

**Figure 8:**
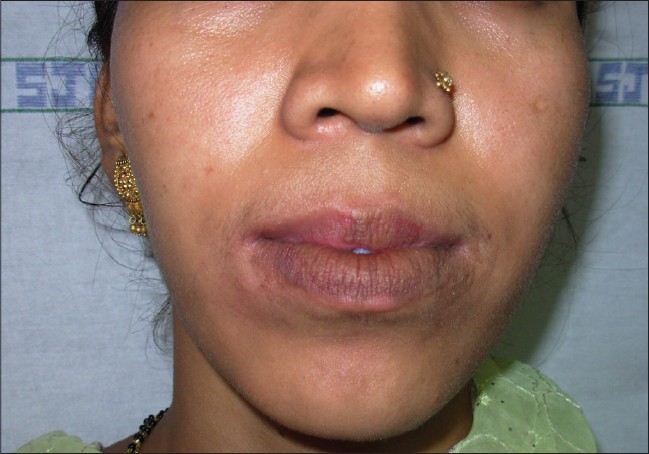
Results of treatment with STSG after 2 months post-operative

Suction blister grafts for small areas and thin split thickness grafts for larger areas, give a good cosmetic outcome on the lips [Figures 7 and 8].[41]

### Areola:

**Figure 9:**
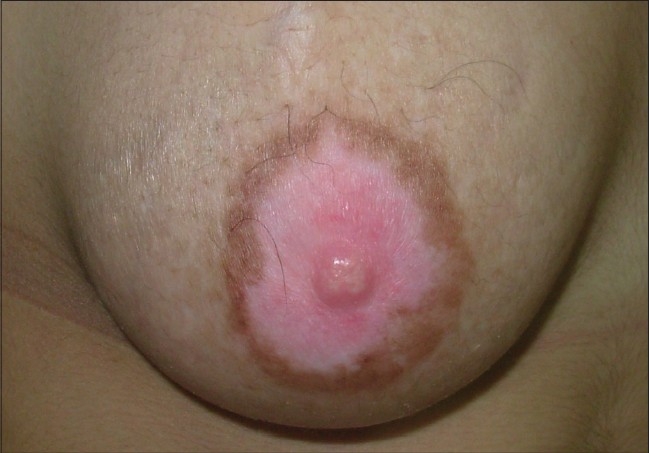
Stable vitiligo of the areola

**Figure 10:**
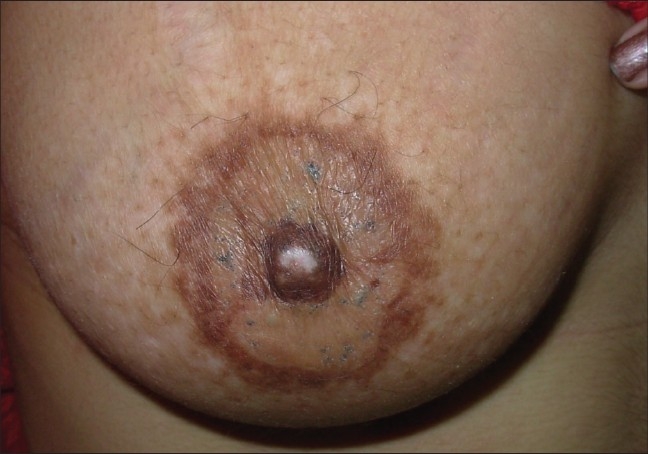
Treatment with STSG

The entire areola should be grafted in order to maintain a uniform color [Figure 9 and 10].

### Acral areas:

The fingers, toes, palms and soles are the most difficult areas to treat and results are not always successful. The possible explanations are absence of hair follicles and thicker epidermis, leading to inadequate dermabrasion and preparation of a good vascular bed. These sites are also more prone to friction and trauma, thus Koebnerization is common.[42] Mini punch grafting is most suitable for these areas. Suction blister grafting or thin split thickness skin grafting is suitable for the dorsal sites. Immobilization using splints is essential for proper graft uptake.

### Genitals:

It is essential to rule out a history of genital herpes, before attempting surgical treatment. It can be a cause of recurrence at the treated site and failure of surgical procedure. Long-term prophylaxis with acyclovir should be given in proven and doubtful cases and the prognosis clearly explained. Suction blister grafts, thin split thickness skin grafts, mini punch grafts and noncultured melanocyte suspensions[43] have been utilized, depending on the size of the vitiligo patch and expertise of the surgeon. Grafts can be secured with cyanoacrylate adhesive or fine stay sutures with 6 0 Prolene.

### Hairy areas:

The hair should not be shaved but plucked out before grafting to delay hair regrowth and lifting up of the graft. Alternatively, a chemical depilatory may be used.

## Mesh Graft

Mesh grafting is a technique where the graft is expanded by making slits in it such that it appears like a mesh. The advantage of this technique is that it allows coverage of large body surface areas with a smaller graft.[44]

### Procedure

The donor site is cleaned and a split thickness graft of 0.0252 inch thickness is obtained either manually or by using a Padgett or Duvals dermatome. The graft is then meshed in an Ampligreffe or Discard-A-pad[45] into any expansion size (1:1, 1:2, 1:4, 1:6). Meshing can be done manually also, by using a sterile blade and making cuts in it. Once the recipient site is cleaned and dermabraded, the graft is transferred and bandaged with saline soaked dressing or sofratulle. The donor site is also dressed using aseptic precautions.

### Follow up

The dressing at the recipient site is removed after a week. Phototherapy is started immediately or after a week.

### Advantages

The meshing of the graft allows larger areas to be covered due to graft expansion. It also allows coverage of areas with variable contours, where a sheet might not adhere well.

### Disadvantages

There is risk of scarring at the donor site. If the graft is thick, then it might lead to beading at the margin. Cosmetic results are inferior as compared to other methods.

## Flip-top Pigment Transplantation

Flip-top technique is a method in which the graft is placed between a flap of epidermis and dermis at the recipient site.[46]

### Procedure

A thin split thickness graft is harvested from either the medical aspect of the upper arm or lateral aspect of the thigh, by using a sterile blade. The grafts are kept moist in saline-soaked gauzes. The recipient site is cleaned and similarly a flap of the epidermis is raised using a sterile blade. One end of the epidermis is left in contact with the dermis and the flap is turned to expose the dermis. The graft is placed with the dermal side of the graft in contact with the dermis and the flap is put back in position to cover the graft. Cyanoacrylate glue is used to secure the graft and the flap. Both the donor and recipient areas are dressed.

### Follow up

The dressing is removed after a week and graft uptake is checked. The patient is started on phototherapy thereafter.

### Advantages

The flap of epidermis acts as a biological dressing. Chances of the graft falling off and secondary infection are less in this procedure. It is inexpensive, easy to perform, and a quick method.

### Disadvantages

Like all dermoepidermal grafts, skill is required to harvest a thin graft or there is beading at the margins.

## Transplantation of Hair Follicles

It has been long considered that repigmentation in vitiligo occurs from the melanocytes in the hair follicle. Hence, hair transplantation in vitiligo patches has been seen to cause repigmentation. Also, tissue grafting leads to repigmentation of the skin, but very rarely of the leucotrichia. Hence, on hair-bearing areas like the eyebrows, scalp, eyelashes, transplantation of hair follicles is required.

### Procedure

A strip of hair is removed from the occipital area of the scalp and cut into smaller pieces, each containing a single hair follicle unit. The hair is then transplanted onto the vitiligo patch. The recipient site is dressed. After a week, the dressing is removed and checked for graft uptake. The patient is started on phototherapy.[47]

### Advantages

It is a good technique for management of leucotrichia. There is no cobblestoning and the color match is good.

### Limitations

It is a cumbersome procedure requiring expertise and time. Scarring occurs at the occipital area from where the graft is taken.

## Conclusion

Vitiligo, characterized by depigmented macules is a common disorder with a high psychosocial impact. Surgical treatment is indicated in resistant stable vitiligo that does not show adequate response to medical therapy. Stability of the disease process is the most important parameter to achieve a successful outcome. Conventional surgical modalities are tissue grafts such as punch grafting, suction blister grafting and split thickness skin grafts. The choice of surgical treatment depends on type of vitiligo, extent and site of lesions, availability of equipment and expertise of the treating surgeon. Split thickness skin grafting is the most successful out of all tissue grafting methods. Recent advances include autologous non-cultured epidermal cell suspensions and cultured melanocyte suspensions or sheets. Hence though surgical treatment of vitiligo is evolving over the years, the basic etiopathogenetic mechanisms need to be elucidated to achieve long term successful results.

## References

[1] Parsad D, Dogra S, Kanwar AJ (2003). Quality of life in patients with vitiligo. Health Quality Life Outcomes.

[2] Falabella R, Gupta S, Olsson MS, Kanwar AJ, Ortonne JP (2007). History and chronology of development of surgical therapies for vitiligo. Surgical management of vitiligo.

[3] Savant SS (2005). Surgical therapy of vitiligo: Current status. Indian J Dermatol Venereol Leprol.

[4] Behl PN (1964). Treatment of vitiligo with homologous thin Thiersch grafts. Curr Med Pract.

[5] Falabella R (1971). epidermal grafting: An original technique and its application in achromic and granulating areas. Arch Dermatol.

[6] Falabella R (1978). Repigmentation of leucoderma by minigrafts of normally pigmented, autologous skin. J Dermatol Surg Oncol.

[7] Falabella R, Escobar C, Borrero I (1989). Transplantation of *in-vitro* cultured epidermis bearing melanocytes for repigmenting vitiligo. J Am Acad Dermatol.

[8] Gauthier Y, Surleve-Bazeille JE (1992). Autologous grafting with non-cultured melanocytes: A simplified method for treatment of depigmented lesions. J Am Acad Dermatol.

[9] Olsson M, Juhlin L (2002). long-term follow-up of leukoderma patients treated with transplants of autologous cultured melanocytes, ultrathin epidermal sheets and basal cell layer suspension. Br J Dermatol.

[10] Kahn AM, Cohen MJ (1998). Repigmentation in vitiligo patients: Melanocyte transfer via ultra-thin grafts. Dermatol Surg.

[11] Kahn AM, Ostad A, Moy RL (1996). Grafting following short pulse CO_2_ laser de-epithelialization. Dermatol Surg.

[12] Das SS, Pasricha JS (1992). Punch grafting as a treatment for residual lesions in vitiligo. Indian J Dermatol Venereol Leprol.

[13] Boersma BR, Westerhof W, Bos JD (1995). Repigmentation in vitiligo vulgaris by autologous minigrafting: Results in nineteen patients. J Am Acad Dermatol.

[14] Olsson M, Juhlin L (2002). long-term follow-up of leukoderma patients treated with transplants of autologous cultured melanocytes, ultrathin epidermal sheets and basal cell layer suspension. Br J Dermatol.

[15] Falabella R (2003). Surgical treatment of vitiligo: Why, when and how. J Eur Acad Dermatol Venereol.

[16] Parsad D, Gupta S (2008). Standard guidelines of care for vitiligo surgery. Indian J Dermatol Venereol Leprol.

[17] Falabella R, Arrunategui A, Barona MI, Alzate A (1995). The minigrafting test for vitiligo: Detection of stable lesions for melanocyte transplantation. J Am Acad Dermatol.

[18] Njoo MD, Das PK, Bos JD, Westerhof W (1999). Association of the Kobner phenomenon with disease activity and therapeutic responsiveness in vitiligo vulgaris. Arch Dermatol.

[19] Laxmisha C, Thappa DM (2005). Reliable site for suction blister induction and harvesting. Indian J Dermatol Venereol Leprol.

[20] Gupta S, Shroff S, Gupta S (1999). Modified technique of suction blistering for epidermal grafting in vitiligo. Int J Dermatol.

[21] Burm JS (2000). Simple suction device for autologous epidermal grafting. Plast Reconstr Surg.

[22] Alexis AF, Wilson DC, Todhunter JA, Stiller MJ (1999). Reassessment of the suction blister model of wound healing: Introduction of a new high pressure device. Int J Dermatol.

[23] Hann SK, Im S, Bong HW, Park YK (1995). Treatment of stable vitiligo with autologous epidermal grafting and PUVA. J Am Acad Dermatol.

[24] Lee DY (2009). The use of suction blisters for recipient site in epidermal grafting: The implications for vitiligo. J Eur Acad Dermatol Venereol.

[25] Lee DY, Park JH, Choi SC, Lee JH (2009). Comparison of recipient site preparations in epidermal grafting for vitiligo: Suction blister and CO_2_ laser. J Eur Acad Dermatol Venereol.

[26] Njoo MD, Westerhof W, Bos JD, Bossuyt PM (1998). A systematic review of autologous transplantation methods in vitiligo. Arch Dermatol.

[27] Gupta S, Kumar B (2003). Epidermal grafting in vitiligo: Influence of age, site of lesion, and type of disease on outcome. J Am Acad Dermatol.

[28] Gupta S, Sandhu K, Kanwar A, Kumar B (2004). Melanocyte transfer via epidermal grafts for vitiligo of labial mucosa. Dermatol Surg.

[29] Nanda S, Relhan V, Grover C, Reddy BS (2006). Suction blister epidermal grafting for management of eyelid vitiligo: Special considerations. Dermatol Surg.

[30] Babu A, Thappa DM, Jaisankar TJ (2008). Punch grafting versus suction blister epidermal grafting in the treatment of stable lip vitiligo. Dermatol Surg.

[31] Khunger N, Gupta S (2007). Thin split-thickness skin grafts for vitiligo. In surgical management of vitiligo.

[32] Kahn A, Cohen M (1995). Vitiligo: Treatment by dermabrasion and epithelial sheet grafting. J Am Acad Dermatol.

[33] Juhlin L, Olsson MJ (1995). Optimal application times of eutectic mixtures of local anaesthetics (EMLA) cream before dermabrasion of vitiliginous skin. Eur J Dermatol.

[34] McGregor IA, McGregor AD (1995). Free skin grafts. Fundamental techniques of plastic surgery.

[35] Ozdemir M, Cetinkale O, Woff R, Wolf R, Kotoyan A, Mat C, Tuzun B (2002). Comparison of two surgical approaches for treating vitiligo: A preliminary study. Int J Dermatol.

[36] Oh CK, Cha JH, Lim LY, Jo JH, Kim SJ, Jang HS (2001). Treatment of Vitiligo with suction epidermal grafting by the use of an Ultrapulse CO_2_ laser with a Computerised Pattern Generator. Dermatol Surg.

[37] Torium DM, O'Grody K, Desai D, Bagal A (1998). Cyanoacrylate Adhesive. Plast Reconstr Surg.

[38] Khunger N (2003). Cyanoacrylate Adhesive in Split thicknes skin Grafts for Resistant Vitiligo- an experience in 50 cases. Presented at 31^st^ National Conference of Indian Association of Dermatologist Veneorologist and Leprologist.

[39] Khunger N, Ghosh S (2007). Recent advances in the surgical treatment of vitiligo. In recent advances in dermatology.

[40] Behl PN, Azad O, Kak R, Srivastava G (1999). Autologous thin Thierschs Grafts in vitiligo: Experience of 8000 cases 50000 grafts (1959-98) with modIfied technique in 198 cases in the year 1997-98. Indian J Derm Venereol Leprol.

[41] Gupta S, Goel A, Kanwar AJ, Kumar B (2006). Autologous melanocytes transfer via epidermal grafts for lip vitiligo. Int J Dermatol.

[42] Mutalik S, Gupta S (2007). Surgical management of acral vitiligo. In surgical management of vitiligo.

[43] Mulekar SV, Al Issa A, Al Eisa A, Asaad M (2005). Genital vitiligo treated by autologous, noncultured melanocyte-keratinocyte cell transplantation. Dermatol Surg.

[44] Srinivas CR, Rai R, Uday Kumar P (2004). Meshed split skin graft for extesive vitiligo. Indian J Dermatol Venereol Leprol.

[45] Sinha M (2005). meshing small skin grafts manually: A simple, quick and effective method using discard-a-pad. Ann Plast Surg.

[46] McGovern TW, Bolognia J, Leffell DL (1999). Flip-top pigment transplantation. Arch Dermatol.

[47] Malakar S, Dhar S, Malakar RS (1999). Repigmentation of vitiligo patches by transplantation of hair follicles. Int J Dermatol.

